# Differential Host Cell Gene Expression and Regulation of Cell Cycle Progression by Nonstructural Protein 11 of Porcine Reproductive and Respiratory Syndrome Virus

**DOI:** 10.1155/2014/430508

**Published:** 2014-02-26

**Authors:** Yan Sun, Dong Li, Sumanprava Giri, Supriya G. Prasanth, Dongwan Yoo

**Affiliations:** ^1^Department of Pathobiology, University of Illinois at Urbana-Champaign, 2001 South Lincoln Avenue, Urbana, IL 61802, USA; ^2^Department of Pathobiology, University of Pennsylvania, 380 South University Avenue, Philadelphia, PA 19104, USA; ^3^Department of Cell and Developmental Biology, University of Illinois at Urbana-Champaign, Urbana, IL 61801, USA

## Abstract

Nonstructural protein 11 (nsp11) of porcine reproductive and respiratory syndrome virus (PRRSV) is a viral endoribonuclease with an unknown function. The regulation of cellular gene expression by nsp11 was examined by RNA microarrays using MARC-nsp11 cells constitutively expressing nsp11. In these cells, the interferon-**β**, interferon regulatory factor 3, and nuclear factor-**κ**B activities were suppressed compared to those of parental cells, suggesting that nsp11 might serve as a viral interferon antagonist. Differential cellular transcriptome was examined using Affymetrix exon chips representing 28,536 transcripts, and after statistical analyses 66 cellular genes were shown to be upregulated and 104 genes were downregulated by nsp11. These genes were grouped into 5 major signaling pathways according to their functional relations: histone-related, cell cycle and DNA replication, mitogen activated protein kinase signaling, complement, and ubiquitin-proteasome pathways. Of these, the modulation of cell cycle by nsp11 was further investigated since many of the regulated genes fell in this particular pathway. Flow cytometry showed that nsp11 caused the delay of cell cycle progression at the S phase and the BrdU staining confirmed the cell cycle arrest in nsp11-expressing cells. The study provides insights into the understanding of specific cellular responses to nsp11 during PRRSV infection.

## 1. Introduction

Porcine reproductive and respiratory syndrome (PRRS) is one of the most significant infectious diseases for the pig industry worldwide and causes severe economic losses [1]. The etiological agent is PRRS virus (PRRSV), which belongs to the family Arteriviridae in the order Nidovirales [2] and possesses a single-stranded positive-sense RNA genome of 15.4 kb in size [3–6]. Two distinct genotypes have been reported for PRRSV: European (type I) and North American (type II) genotypes [7, 8]. The PRRSV genome contains 10 open reading frames (ORFs) including the newly identified ORF5a [9, 10]. ORF1a is translated to produce the PP1a polyproteins, but ORF1b is expressed as a fusion with ORF1a by ribosomal frameshifting and produces the PP1a/b fusion polyproteins. PP1a and PP1a/b are cotranslationally processed into 14 cleavage products. These products are nonstructural proteins (nsps) that are believed to participate in viral genome replication and subgenomic mRNA transcription [11–13]. Of these, nsp11 is a 223 amino acid protein and contains a nidovirus-specific domain, termed NendoU, in the C-terminal region. NendoU is known to contain an endoribonuclease activity and consists of two subdomains, A and B [4, 14–16]. Mutational studies using equine arteritis virus (EAV) nsp11, which is a homolog of PRRSV nsp11, show that three enzymatically catalytic sites reside in subdomain A, while two aspartic acids in subdomain B are responsible for the overall protein structure [16]. In EAV, nsp11 plays a key role in viral RNA synthesis and thus it may also be essential for PRRSV replication. Recently, PRRSV has been shown to modulate type I IFN response [17] and nsp11 has been suggested to participate in the modulation of IFN response [18].

Cellular transcriptional profiles during PRRSV infection have been studied to some extent [19, 20]. However, such studies do not identify specific viral proteins responsible for gene expressions changes, and thus the present study was conducted to understand the specific cellular response to nsp11 in cells stably expressing the protein using RNA microarrays. Based on the microarray data, five major cellular pathways were identified to be regulated by nsp11, and of the five pathways the cell cycle pathway was examined. We provide the evidence that PRRSV nsp11 protein participates in modulating the cell cycle progression at the S phase.

## 2. Materials and Methods

### 2.1. Cells

MARC-145 is a subcloned cell line of MA-104 which was derived from African green monkey kidney [21]. MARC-145 is the only established cell line permissive for PRRSV replication and thus widely used for the study of PRRSV *in vitro*. MARC-145 and MARC-nsp11 cells were maintained in Dulbecco's modified Eagle's medium (DMEM; Mediatech Inc., Manassas, VA, USA) containing 10% heat-inactivated fetal bovine serum (FBS; HyClone, Logan, UT, USA) in a humidified incubator with 5% CO_2_ at 37°C.

### 2.2. Plasmids, Antibodies, and Chemicals

The nsp11 coding sequence was PCR-amplified from the FL12 strain of PRRSV and was inserted into the retroviral expressing vector pLNCX2 (Clontech) and mammalian expression vector pXJ41 with a FLAG tag at its N-terminus using the following primers: forward 5′-AAACTCGAGGCCACCATGGGGTCGAGCTCCCCGCTCCC-3′ and reverse 5′-GCGGCCGCTTACTTATCGTCGTCATCCTTGTAATCTTCAAGTTGAAAATAGGC-3′. The translation initiation and termination codons were added to the nsp11 coding sequence. The anti-FLAG monoclonal antibody (MAb M2, Sigma) and the anti-BrdU antibody were purchased from Sigma (St. Louis, MO, USA). Bromodeoxyuridine (5-bromo-2′-deoxyuridine, BrdU) is a synthetic nucleoside that is an analog of thymidine and is commonly used in the detection of proliferating cells. Polyinosinic:polycytidylic (poly [I:C]) as a double-stranded RNA analog was purchased from Sigma. A donkey anti-rabbit antibody conjugated with Texas Red and a goat anti-mouse antibody conjugated with FITC were purchased from Invitrogen (Carlsbad, CA, USA). The nsp11-specific rabbit antibody was generated in our laboratory using recombinant proteins described as follows.

### 2.3. Recombinant Protein Preparation

Since wild-type nsp11 seemed to be toxic in *E. coli* [15], the NendoU nsp11 mutant (nsp11-K3779A), was subcloned into the *E. coli* expression vector pET-28a+ with the His-tag at both termini, and this plasmid was transformed into *E. coli* BL21. A 5 mL overnight culture was started using LB broth containing ampicillin (1 *μ*L/mL) by inoculating with transformed bacteria at 37°C with vigorous agitation. In the following morning, 500 mL of 2xYT (16 g of tryptone, 10 g of yeast extract, and 5 g of NaCl per L) containing ampicillin was inoculated with the 5 mL overnight culture. The culture was incubated at 37°C for approximately 4 h and when the optical density at 600 reached 0.6–0.8, protein expression was induced by adding IPTG up to 1 mM concentration. The culture was incubated for additional 2 h. Cells were pelleted by centrifugation at 7700 ×g for 10 min at 4°C. The cells were resuspended in 12.5 mL of STE (5 mL of 1 M Tris-HCl, pH 8.0, 150 mL of 0.5 M NaCl and 1 mL of 0.5 M EDTA, pH 8.0 in 500 mL) containing aprotinin (1–10 *μ*g/mL) and PMSF (1 mM) and pelleted again at 7700 ×g for 10 min. The cells were resuspended again in 12.5 mL of 1X PBS, and DNase I (20 *μ*g/mL) and lysosome (200 *μ*g/mL) were added and treated for 1 h. Then, DTT was added to make a final concentration of 5 mM and incubate 5 min on ice. 20% of Sarkosyl solution was additionally added to a final concentration of 0.5%, followed by sonication to sheer the genomic DNA at the setting scale of 4 for 10 s at least three times (Soniprep 150; Sanyo Gallenkamp PLC, Leicester, UK). After sonication, the samples were centrifuged at 12,000 rpm for 30 min (J2-21; Beckman Coulter, Brea, CA, USA), and supernatants and pellets were collected separately and subjected to SDS-PAGE individually to determine the presence of nsp11-K3779A protein for each fraction. Nsp11 was purified from the supernatants and concentrated to 1 mg/mL using the HisTrap column according to the manufacturer's instruction (GE Healthcare Life Sciences, Piscataway, NJ, USA). A total of 2 mg of nsp11 was used to immunize a rabbit 5 times at 2-week intervals, intramuscularly using Freund's incomplete and complete adjuvants, and an anti-PRRSV-nsp11 rabbit serum was generated (Immunological Research Center, University of Illinois, Urbana, IL, USA). The specificity of the antiserum was determined by immune-blot and immunofluorescence using PRRSV-infected MARC-145 cells.

### 2.4. Establishment of nsp11-Expressing Cells (MARC-nsp11)

MARC-145 cells were transduced with the nsp11 gene using the retroviral gene transfer system (Clontech). Briefly, 0.5 *μ*g of the pLNCX2-FLAG-nsp11 plasmid was cotransfected with pVSV-G into the pantropic packaging cell line GP2-293 to produce infectious lentivirus containing the PRRSV nsp11 gene. After 48 h of incubation, culture supernatants were collected and used to infect MARC-145 cells. Nsp11 gene-integrated cells were selected using 1 mg/mL of G418 (Invitrogen) for approximately 2 weeks with fresh G418 every 4 days. When the majority of cells has died, G418-resistant cell colonies were picked using cloning cylinders and were amplified as putative nsp11-expressing cells. Seven clones were initially selected and individually amplified. One clone was chosen and designated as MARC-nsp11 for subsequent studies.

### 2.5. PCR, RT-PCR, and Quantitative PCR

For PCR, cellular DNA was extracted from MARC-nsp11 cells using QIAamp DNA kit (Qiagen) and PCR was performed to determine the nsp11 gene integration. For reverse transcription (RT), total cellular RNA was extracted using Trizol (Invitrogen) and was treated with RQ1 RNase-free DNase I (Promega) followed by RT using the nsp11-specific reverse primer and PCR using the primer set as described above. Quantitative (q) PCR was performed using ABI Prism 7000 Sequence Detection System and Software (Applied Biosystems) in a final volume of 25 *μ*L containing 2.5 *μ*L of cDNA synthesized from the RT reaction, 2.5 pmol of each primer, 12.5 *μ*L of SYBR Green Master Mix (Applied Biosystems), and 5 *μ*L of water. The primer sequences were designed using Primer 5.0 Software (Invitrogen) or obtained from previous reports (Table 2). The amplification parameters were 40 cycles of two steps, each step comprised of heating at 95°C and extension at 60°C. The final mRNA levels of target genes were normalized using GAPDH as a house keeping gene.

### 2.6. Immunoprecipitation

Typically, 100 *μ*L of total cell lysates was incubated with 1 *μ*L of the anti-nsp11 rabbit serum at 4°C overnight. Reactions were incubated with Protein A Sepharose beads (GE Healthcare) at 4°C for 4 h. Following centrifugation for 5 min, supernatants were aspirated and washed with the lysis buffer twice. The beads were mixed with the loading buffer, boiled, and subjected to 12% SDS-PAGE followed by transfer to Immobilon-P membrane (Millipore). After blocking membranes with 5% skim milk powder dissolved in TBS-T (10 mM Tris-HCl [pH 8.0], 150 mM NaCl, 1% Tween 20) for 1 h at room temperature, membranes were incubated with the anti-FLAG antibody in TBS-T containing 5% skim milk powder at 4°C overnight. After 5 washes with TBS-T, membranes were incubated with a horseradish peroxidase-conjugated anti-mouse antibody for 1 h at room temperature. Membranes were washed 5 times again and proteins were visualized using the ECL detection system (Thermo, Minneapolis, MN, USA).

### 2.7. Dual Luciferase Reporter Assays

Double-strand RNA stimulation was conducted using poly (I:C). For nsp11-gene transfection, MARC-145 cells were seeded in 12-well plates and per well, 0.05 *μ*g of pRL-TK, 0.5 *μ*g of pIFN-*β*-luc, pIRF3-luc or pPRDII-luc, and 0.5 *μ*g of pXJ41-FLAG-nsp11 were cotransfected using Lipofectamine 2000 according to the manufacturer's instruction (Invitrogen). For MARC-nsp11 cells, pRL-TK and each of the three reporter plasmids were cotransfected with the same amount as that of the nsp11 gene transfection. Twenty-four hours after transfection, 0.5 *μ*g of poly (I:C) was transfected into cells for 16 h. Cells were lysed using the passive lysis buffer (Promega), and supernatants were measured for luciferase activities using the Dual Luciferase Reporter Assay System (Promega) in the luminometer (Wallac 1420 Victor multilabel counter, Perkin Elmer, Waltham, MA, USA).

### 2.8. RNA Microarray Design and Data Analyses

MARC-145 and MARC-nsp11 cells were seeded one day prior to experiments and total cellular RNAs were extracted using Trizol (Invitrogen) and purified by RNeasy mini kit (Qiagen). The quantity and quality of RNA were determined using an Align 2100 bioanalyzer (Agilent Technologies, Palo Alto, CA). The RNA samples were then subjected to microarray using Human Gene 1.0 ST arrays (Affymetrix UK Ltd., High Wycombe, UK) at Keck Biotechnology Center (University of Illinois, Urbana, IL). The microarray was repeated twice in triplicate each. For data analyses, quality control assessments, data processing, and statistical analyses were conducted using the package from the Bioconductor project [22] as indicated below. The Affymetrix's Human Gene 1.0 ST array contained probes to interrogate 253,002 exons representing 28,536 annotated genes. Comparisons were made either on the exon-level to investigate alternative splicing or on the whole gene-level to summarize all transcripts of the gene. The individual probe values were background-corrected, normalized, and summarized into one value at both the exon- and gene-levels using the robust multiarray average (RMA) algorithm available from the oligo packages [23]. Testing for differential gene expressions between MARC-145 cells and MARC-nsp11 cells was conducted separately at the exon- and gene-levels by fitting a linear model including a term to account for the separate processing batches using the Linear Models for Microarray Data (Limma) package [24, 25]. The criteria for significance varied for the exon- and gene-levels. At the exon-level, the criteria were at least a 2-fold change and a raw *P* value <0.02, resulting in 8,693 significant exons. At the gene-level, the Limma model was fit and raw *P* values were calculated using all genes on the array, but the correction for multiple hypothesis testing using the FDR (false discovery rate) method [26] was done for only the 9,241 genes that varied in expression across all the samples of at least a 1.5-fold change. The criteria used to select significant genes within the filtered database for upregulation and downregulation were FDR *P* value <0.1 and fold change >2 or <−2, respectively.

### 2.9. Flow Cytometry and Cell Cycle Analysis

Identical numbers of MARC-145 cells and MARC-nsp11 cells were seeded and grown for 24 h in DMEM containing 10% FBS. For flow cytometry, cells were collected by trypsinization, washed with PBS, and resuspended in cold PBS to 1 × 10^6^ cells per mL. The cell suspension was added dropwise to an equal volume of cold ethanol with continuous agitation. After overnight incubation at 4°C, its cellular DNA was stained with 10 *μ*g/mL propidium iodide (PI) prepared in PBS containing 0.1% Triton X-100 and 10 *μ*g/mL RNase A (Roche) for 30 min at room temperature in the dark. Samples were then analyzed by flow cytometry (BD AccuriC6, BD Accuri Cytometers, Ann Arbor, MI, USA), and the data were analyzed using FACS Express software supplied from Keck Biotechnology Center (University of Illinois, Urbana, IL, USA).

### 2.10. BrdU Incorporation and Immunofluorescence Assay

DNA synthesis in proliferating cells was determined using (BrdU) bromodeoxyuridine incorporation since its incorporation to DNA occurs during the S phase. Cells were seeded on cover slips at a density of 1 × 10^5^ cells/cover slip (10 mm × 10 mm) and allowed to rest for 24 h. The medium was removed and cells were incubated for a 10 min, 20 min, or 24 h pulse in the BrdU labeling medium. For the 10 min and 20 min pulses, 10 *μ*M of BrdU was applied, and for the 24 h pulse 100 nM of BrdU was applied. After BrdU incubation, cells were fixed in 2% paraformaldehyde in PBS for 15 min and washed with PBS three times. Cells were then permeabilized with 0.5% Triton X-100 for 7 min on ice followed by blocking with 1% normal goat serum (NGS) in PBS three times, 10 min each. To observe the nuclei of cells, an anti-lamin (1 : 200) rabbit antibody was used as the primary antibody for 1 h in PBS containing 1% NGS, and cells were incubated with a donkey anti-rabbit antibody conjugated with Texas-red (1 : 1000) for 30 min. Followed by washing four times, 5 min each, 2% paraformaldehyde was used again to fix the bound antibodies. Cells were then incubated with 4N HCl for 25 min at room temperature to denature DNA and to allow the BrdU antibody to recognize the incorporated BrdU in the nuclei. After three 10 min washes with PBS and two 10 min washes with 1% NGS in PBS, an anti-BrdU monoclonal antibody (1 : 500) was applied to cells for 90 min. Cells were then incubated with a goat anti-mouse antibody conjugated with FITC (1 : 600) for 30 min. Washing with PBS was applied four times after every incubation with antibodies. The cover slips were mounted on microscope slides in the mounting buffer and visualized using a Zeiss Axio Imager z1 fluorescence microscope (Carl Zeiss Inc.) equipped with Chroma filters (Chroma Technology). Images were collected using AxioVision Software (Zeiss) and Hamamatsu ORCA cooled CCD camera. The BrdU-incorporated cells and a total number of lamin-positive cells were counted for both MARC-145 and MARC-nsp11 cells, and the % of BrdU incorporation was calculated using the formula as follows: % BrdU incorporation = (number of double-positive cells for BrdU and lamin)/(200 lamin-positive cells) × 100. The cell counting areas were selected randomly on slides and 200 lamin-positive cells were counted.

## 3. Results

### 3.1. Establishment of MARC-nsp11 Cells Stably Expressing nsp11 Protein

**Figure 1:**
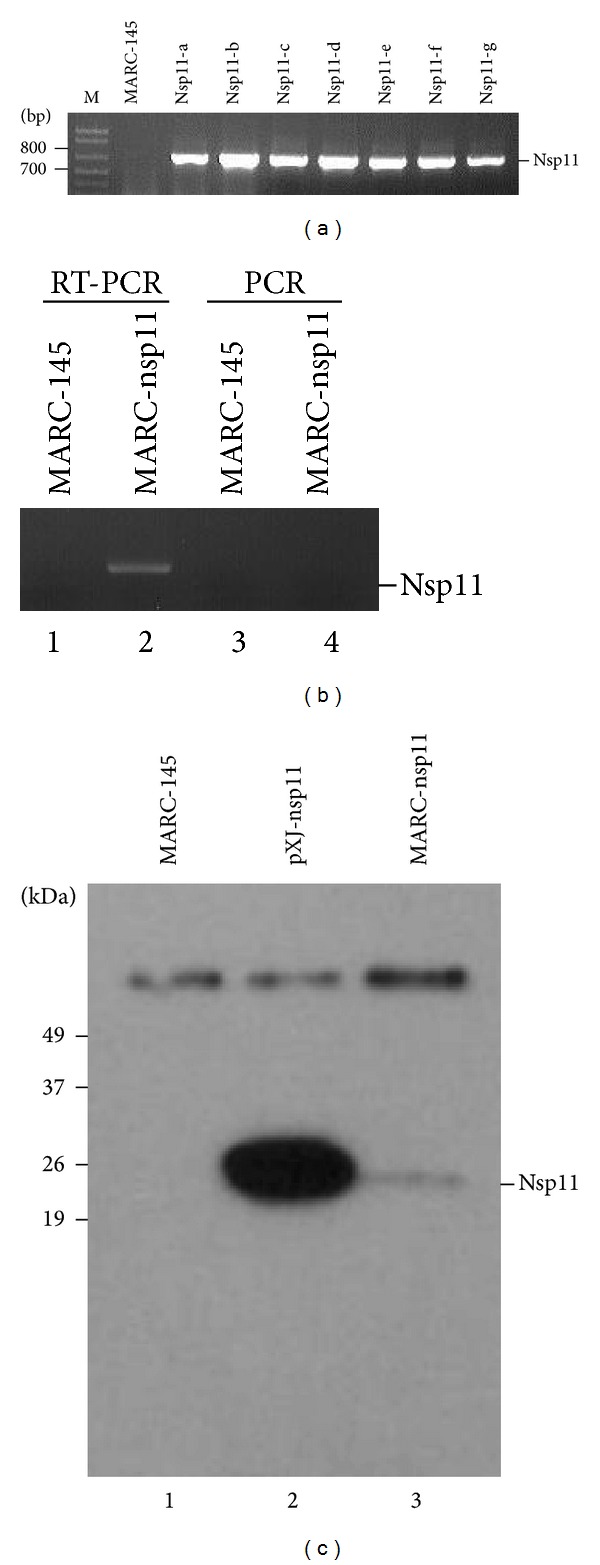
Establishment of MARC-nsp11 cells stably expressing PRRSV nsp11. (a) Incorporation of nsp11 gene in cellular DNA and identification of cell clones. A total of 7 clones, designated nsp11-a through nsp11-g, were obtained and screened for nsp11 sequence by PCR. Cellular DNA was extracted and PCR was performed using the primers described in Materials and Methods. The nsp11-a clone was chosen to conduct RT-PCR (b) and immune-blot (c) and designated as MARC-nsp11 cells. (b) Total cellular RNA was extracted from MARC-145 and MARC-nsp11 cells and subjected to DNase I treatment followed by RT-PCR or PCR. (c) Cell lysates were prepared from MARC-145 (lane 1), nsp11-gene transfected MARC-145 (lane 2), and MARC-nsp11 (lane 3) cells and were incubated with Protein A Sepharose beads and anti-rabbit nsp11-specific polyclonal Ab, followed by immunoblot using anti-FLAG monoclonal Ab.

To study the regulatory role of PRRSV nsp11 in cellular processes, a cell line was established to constitutively express the nsp11 protein. MARC-145 was used as the parental cell line, since it is one of only few cell lines permissive for PRRSV infection. MARC-145 cells were infected with the lentivirus containing the nsp11 gene from PRRSV strain FL12 and G418 (neomycin)-resistant cells were selected. A total of seven drug-resistant clones were obtained and they were individually propagated for analyses. Cellular DNA was extracted from each clone, and PCR was conducted to determine gene integration. All clones were PCR-positive for nsp11 (Figure 1(a)), and cell clone “a” was chosen for further characterization and designated MARC-nsp11. The chosen cell clone was examined for nsp11 mRNA by RT-PCR using primers indicated in Table 2, and a 660 bp fragment was specifically amplified (Figure 1(b)). The nsp11 protein expression was also determined by immunoprecipitation using a rabbit anti-nsp11 antibody. A 26 kD protein was specifically identified in MARC-nsp11 cells at a low level (Figure 1(c), lane 3), and the same size protein was identified in nsp11 gene-transfected cells (Figure 1(c), lane 2), demonstrating the expression of nsp11 in these cells.

### 3.2. Suppression of Type I IFN Induction by nsp11

**Figure 2:**
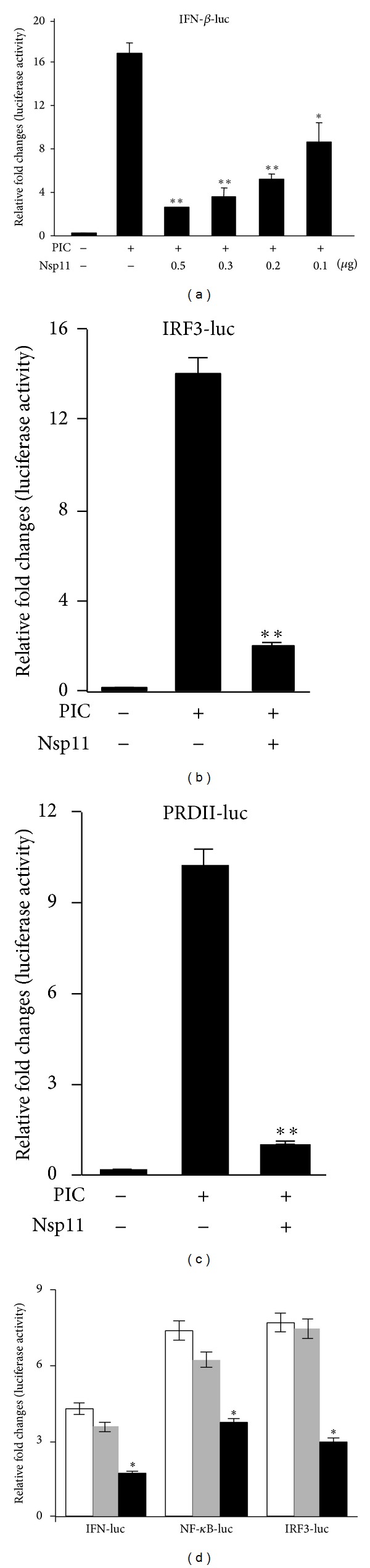
Suppression of type I IFN induction by PRRSV nsp11 in gene-transfected MARC-145 cells (a, b, and c), and stably-expressing MARC-nsp11 cells (d). (a) MARC-145 cells were seeded in 12-well plates and transfected with pXJ41 (0.5 *μ*g) or pXJ41-Flag-nsp11 plasmid of 0.5, 0.3, 0.2, or 0.1 *μ*g, together with pRL-TK (0.05 *μ*g) and pIFN-*β*-luc (0.5 *μ*g) reporter. After 24 h, cells were transfected with 0.5 *μ*g/mL of poly (I:C) for 16 h and harvested for luciferase assay (Promega). (b) and (c) MARC-145 cells were cotransfected with either pIRF3-luc (0.5 *μ*g) or pPRDII-luc (0.5 *μ*g) or pXJ41-Flag-nsp11 (0.5 *μ*g) or pXJ41 (0.5 *μ*g), along with pRL-TK. After 24 h, cells were transfected with 0.5 *μ*g/mL of poly (I:C) for 16 h and harvested for luciferase assay (Promega). The experiments were conducted in duplicate and repeated three times, and the overage values were depicted. The fold change was calculated by (PIC+)/(PIC−) for each sample. The negative control represents the basal level luciferase activity. The values from nsp11 gene-transfected samples were compared with those of poly (I:C) stimulation, and the data were analyzed using 2-tail *t*-test. One star (∗) represents *P* < 0.01 and two stars (∗∗) represent *P* < 0.005. (d) MARC-145 or MARC-nsp11 cells were cotransfected with pIFN-*β*-luc, pIRF3-luc, or pPRDII-luc and pRL-TK. MARC-145 cells were transfected with the pLNCX2 empty vector as a negative control. All samples were treated and processed as described above. Luciferase assays were conducted in duplicate and repeated three times. One star (∗) represents *P* < 0.05. White bars represent MARC-145 cells, grey bars represent the pLNCX2 retrovirus expression vector-transfected MARC-145 cells, and black bars represent MARC-nsp11 cells.

PRRSV nsp11 contains the NendoU domain which is a common motif associated with an endoribonuclease activity for viruses in the order Nidoviridae [14, 15, 27]. Furthermore, PRRSV nsp11 has recently been suggested as a potential type I interferon (IFN) regulator [17, 18]. Thus, the regulatory function of nsp11 for IFN induction was first examined in MARC-nsp11 cells and in MARC-145 cells transfected with the nsp11 gene. Cells were transfected with the pIFN-*β*-luc reporter plasmid and stimulated with poly (I:C) to examine the IFN induction (Figure 2(a)). While the cells transfected with the empty vector pXJ41 showed an efficient induction of luciferase activity of up to ~16-fold after stimulation, nsp11-expressing cells exhibited a strong suppression of the activity down to ~4-fold at the most (*P* < 0.01). The nsp11-mediated IFN suppression was dose-dependent (Figure 2(a)).

IFN expression is tightly regulated by IRFs (interferon regulatory factors), nuclear factor (NF)-*κ*B, and activator protein (AP)-1 transcription factors. Among these, IRFs and NF-*κ*B are major players regulating the formation of IFN enhanceosome and the IFN-*β* production, and thus we first examined the IFN regulatory activities of nsp11 in MARC-145 cells by gene transfection using pIRF3-luc and pPRDII-luc reporter plasmids. pIRF3-luc contains 4 copies of the IRF3-binding sequence, while pPRDII-luc contains 2 copies of the NF-*κ*B binding sequence upstream of the luciferase gene. When cells were stimulated, the IRF3 reporter activity was increased by ~14-fold (Figure 2(b)). In the presence of nsp11, however, the IRF3 activity was decreased by 7-fold (*P* < 0.005) compared to the activity in the absence of nsp11 (Figure 2(b)). Similarly, the NF-*κ*B reporter activity was increased by approximately 10-fold after stimulation, but in the presence of nsp11, this activity was deceased by 2-fold (*P* < 0.005) compared to the activity in the absence of nsp11 (Figure 2(c)). These results show the suppression of IRF3 and NF-*κ*B induction by nsp11.

To examine whether MARC-nsp11 cells expressing nsp11 were biologically active, the IFN-*β*, IRF3, and NF-*κ*B activities were determined after stimulation with poly (I:C) using the corresponding reporter constructs (Figure 2(d)). MARC-nsp11 cells (black bars) showed the decrease of luciferase activities compared to those of empty vector-transfected (gray bars) or mock-transfected (white bars) MARC-145 cells. The suppressive activities in MARC-nsp11 were less markedly than those in gene-transfected cells and this was probably due to the lower level expression of nsp11 in MARC-nsp11 cells. The reporter activities of IFN-*β*, IRF3, and NF-*κ*B were reduced by ~2, 2, and 2.5-fold, respectively (*P* < 0.05). This indicates that nsp11 in MARC-nsp11 cells was biologically active and retained the modulatory activity for IFN induction.

### 3.3. Transcriptome Analysis in MARC-nsp11 Cells

To examine the transcription regulation of host cells by nsp11, an RNA microarray was conducted in MARC-nsp11 cells using human gene exon chips. These chips contained 253,002 exons from 28,536 annotated genes. After microarray analyses, genes were filtered by fold changes greater than 1.5, and 9,241 genes were initially identified to have been altered, among which 66 and 104 cellular genes were upregulated and downregulated, respectively, under the criteria of a fold change of 2 or greater and a false discovery rate (FDR) of 10%. Based on the Database for Annotation, Visualization, and Integrated Discovery (DAVID), 79 of the significantly regulated genes were placed into 17 categories, some of which shared the common function. According to their functional correlations, the functional groups were summarized into five major cellular pathways that were regulated by nsp11: histone-related proteins, cell cycle and DNA replication pathways, MAPK signaling pathways, ubiquitin-proteasome pathways, and complementary pathways (Table 1).

**Figure 3:**
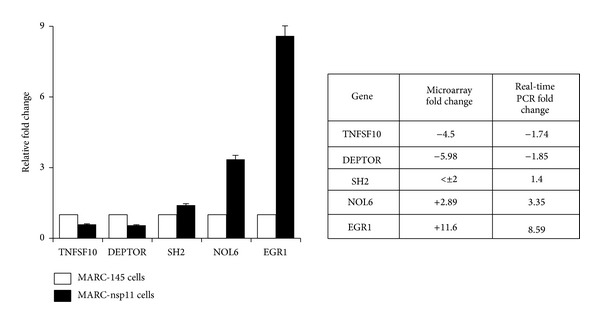
Confirmation of differential gene expression by RT-qPCR in MARC-nsp11 cells. The same preparations that were used for RNA microarray were used for RT-qPCR. For quantitative PCR, two genes (NOL6 and EGR1) were chosen to represent upregulated genes by nsp11, and TNFSF10 and DEPTOR were chosen as the downregulated genes in the microarray assays (Table 2). SH2 was chosen as a nonregulated gene. Bars illustrate the differential gene expression determined by RT-qPCR. White bars represent MARC-145 cells and black bars indicate MARC-nsp11 cells. Numbers in the table show the fold changes.

For validation of the fold changes in the gene expression profiles, five genes (TNFSF10, DEPTOR, SH2, NOL6, and EGR1) were chosen according to their fold changes, and RT-qPCR was conducted. NOL6 and EGR1 were chosen to represent the group of upregulated genes, and TNFSF10 and DEPTOR were chosen to represent the group of downregulated genes, while SH2 was chosen as an unregulated gene. The results from RT-qPCR for these genes were in good agreement with their fold changes in the microarray and confirmed the fold change profiles for differential gene expression (Figure 3).

### 3.4. Regulation of Histone-Related Functions, Complement, MAPK Signaling, and Proteasome Pathways

Seventeen histone-related genes were found to be upregulated, whereas three genes (C1S, C1R, and C3) in the complement system were downregulated (Table 1). C1S and C1R were responsible for the activation of the classic pathway of the complement system. C1R is autoactivated and then cleaves C1S for activation. Activated C1S cleaves C4 and C2, resulting in the activation of C3-convertase complex [28]. C3 is a central molecule in the complement system whose activation is essential for all the important functions performed by this system [29]. The downregulation of C1S, C1R, and C3 suggests the possible suppression of the complement system by nsp11.

Six genes (DUSP1, DUSP6, FOS, MYC, JUN, and SRF) related to the MAPK signaling pathways were found to be upregulated, and five genes in the proteasome pathways were found to be regulated, among which SUMO1 and SNCA were downregulated and PSMD3, PSMB10, and PSMA7 were upregulated (Table 1). DUSPs regulate the cellular localization and activity of MAPK which functions in the negative feedback loop of ERK regulation [30]. DUSP1 dephosphorylates ERK in the nucleus and allows its trafficking to the cytoplasm [31], while DUSP6 causes the cytoplasmic retention of ERK2 [31]. C-Jun/AP-1 and c-Fos genes were also upregulated in our study, which can be activated by JNK and p38 MAPK [32]. For the proteasome pathways, three (PSMD3, PSMB10, and PSMA7) out of five are proteasome subunits, and their upregulation suggests an enhanced effect on the proteasomal pathways by nsp11. SUMO1 (small ubiquitin-like modifier 1) has multiple functions by attaching itself to substrates referred to as sumoylation. After sumoylation, protein may undergo degradation through the proteasome [33, 34].

### 3.5. Delay of Cell Cycle by nsp11

The microarray data suggested the regulation of cell cycle and DNA replication pathways by nsp11. A total of 10 genes related to these pathways were found to be regulated (Table 1). Among these, cell division cycle 45 (CDC45)-like and CDC25 homolog A are proteins controlling the cell cycle progression [35–37], whereas minichromosome maintenance complex 2 (MCM2), MCM4, and MCM5 are helicase components regulating DNA replication [38].

**Figure 4:**
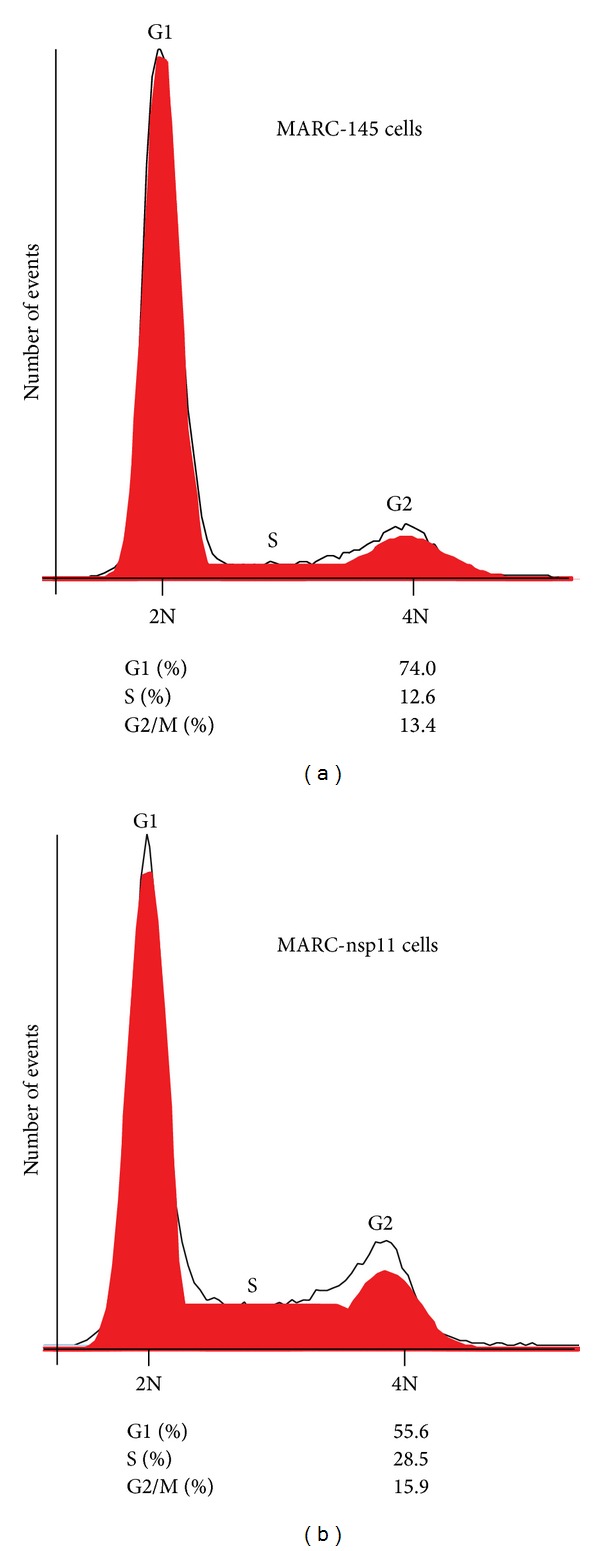
Flow cytometric analysis for MARC-nsp11 cells. A total of 1 × 10^6^ MARC-145 or MARC-nsp11 cells were seeded in 60 mm-diameter dishes and cultivated for 24 h. Cells were gently harvested by trypsinization and fixed with cold ethanol. Cells were stained with 10 *μ*g/mL of propidium iodide (PI) and subjected to flow cytometry. Shown is the PI trace (red areas) indicating the proportion of cells with the 2N and 4N DNA contents. The red areas present relative percentages. G1, first gap period; G2, second gap period; S, DNA synthesis and chromosome replication.

The MCM2-7 complex is assembled on the eukaryotic chromosomes during the G1 phase of a cell cycle, which is then activated during the S phase by MCM10, CDC45, and the GINS complex [39]. The regulation of MCMs and CDC genes suggests that nsp11 may perturb the normal host cell cycle. To examine this possibility, identical numbers of MARC-nsp11 cells and MARC-145 cells were seeded on plates, and 24 h later cells were collected for DNA staining and flow cytometry. In two independent experiments, the MARC-nsp11 cells at the S phase constituted 28.5% (Figure 4(b)) as compared to 12.6% for MARC-145 cells (Figure 4(a)), which was more than a 2-fold increase for nsp11-expressing cells indicating that MARC-nsp11 cells were accumulating at the S phase by 24 h.

**Figure 5:**
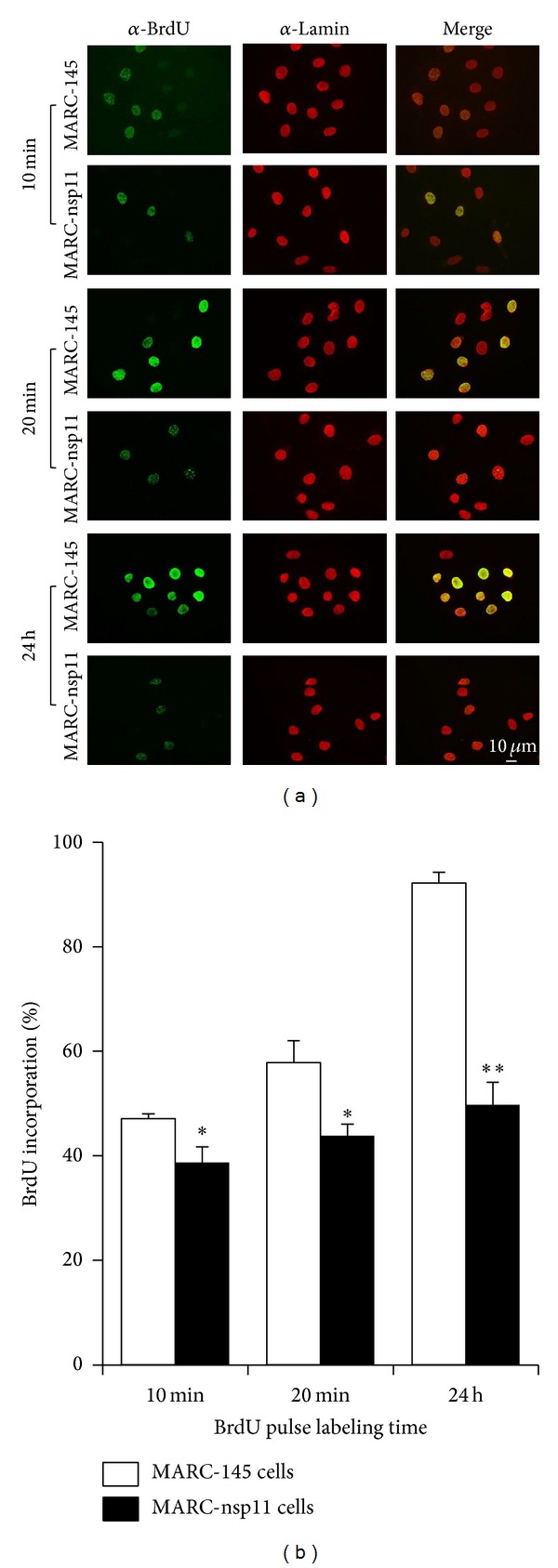
BrdU incorporation and DNA synthesis in MARC-nsp11 cells. (a) Cells were labeled with BrdU and stained to determine the newly synthesized cellular DNA at the S phase. Cells were pulse-labeled with 10 *μ*M of BrdU for 10 min and 20 min, or 100 nM for 24 h. Cells were fixed with 2% paraformaldehyde for 15 min and permeabilized with 0.5% Triton X-100 followed by staining with an anti-lamin antibody (shown in the red color). Cells were then incubated with 4N HCl to denature the DNA, and stained for DNA-incorporated BrdU using an anti-BrdU antibody (shown in the green color). The scale bar in white indicates 10 micron. (b) BrdU incorporation rates in MARC-nsp11 cells. A total of 200 lamin-positive cells were counted, and of the 200 cells BrdU-positive cells were counted. The BrdU incorporation rates were then calculated using the following formula: (number of double-positive cells for BrdU and lamin)/(200 lamin-positive cells) × 100. The experiments were repeated 4 times and the results were presented as the arithmetic means ± standard error (*n* = 4). One star (∗) represents *P* < 0.005 and two stars (∗∗) represent *P* < 0.001. MARC-145 cells are indicated in unfilled white bars and MARC-nsp11 cells are indicated in black bars.

To examine the nature of DNA accumulation at the S phase by nsp11, cells were pulsed-labeled for 10 min, 20 min, or 24 h with BrdU and stained for BrdU incorporation and lamins. BrdU is a nucleotide analog and thus can be incorporated into replicating DNA, whereas lamin proteins are major architectural proteins of the nuclear lining inside the nuclear membrane in cells. Thus, all cells are anticipated to be stained with an anti-lamin antibody, whereas only cells synthesizing new DNA in the S phase are presumed to be stained with an anti-BrdU antibody. A short pulse of 10 min or 20 min would detect BrdU incorporation in a single cell cycle, whereas a longer time incubation of 24 h would detect multiple cell cycles and thus the majority of normal cells would be positive for BrdU staining (Figure 5(a)). A total of 200 lamin-positive cells (in red) were randomly chosen for each slide, and BrdU positive cells (in green) out of the lamin-positive cells were counted to determine BrdU incorporation rates using the formula described in Materials and Methods. MARC-nsp11 cells exhibited less numbers of BrdU-positive cells after the 10 min and 20 min pulses compared to those of MARC-145 cells (Figure 5(a)), and their BrdU incorporation rates dropped from 47.07% (white bar) to 38.07% (black bar) (*P* < 0.005) and from 57.8% (white bar) to 44% (black bar) (*P* < 0.005), respectively (Figure 5(b)). After 24 h of labeling, a greater reduction of BrdU staining was observed for MARC-nsp11 cells, where the percentage of BrdU incorporation decreased from 92% (while bar) to 49.73% (black bar) (*P* < 0.001; Figure 5(b)). The intensity of BrdU staining in MARC-nsp11 cells was also significantly reduced after the 24 h pulse compared to that of MARC-145 cells (Figure 5(a)), demonstrating the substantial suppression of DNA synthesis by nsp11. Both flow cytometry and BrdU staining data indicate that nsp11 slows down the cell cycle progression through the S phase.

## 4. Discussion

In the present study, MARC-nsp11 cells were established to constitutively express PRRSV nsp11, and an RNA microarray was conducted in these cells to study differential transcription responses to nsp11. The microarray studies identified 170 differentially regulated cellular genes with the threshold of 2. Of these, 104 genes were downregulated and 66 genes were upregulated, and many of these genes were able to be placed according to their functional relevance into 5 different pathways: histone-related proteins, cell cycle and DNA replication, MAPK signaling, ubiquitin-proteasome, and the complement system. Compared to previous studies [19, 20], the genes identified in our study were fewer in number and less in diverse. This is probably because the regulated genes identified in our study were exclusively nsp11-specific, whereas the genes in the previous studies were responders to the entire spectrum of viral proteins. Thus, nsp11-regulated genes were mostly included in the previously identified genes. Zhou et al. [20] also showed that the genes relevant to cell cycle and DNA replication were regulated by highly pathogenic (HP)-PRRSV in PAMs. Chromosome organizing proteins were also regulated by nsp11, and proteins regulating the complement system for tissue remolding and inflammation were also found in our study. c-Jun and c-Fos are two effectors of the MAPK signaling pathway, and they were specifically upregulated during PRRSV infection [19]. PRRSV-mediated activation of the MAPK signaling pathway and the increase of JNK and p38 phosphorylation have recently been demonstrated [40], which is also in support of our findings.

Of the possible pathways regulated by nsp11, the cell cycle pathway was chosen and explored further. It appears that the cell cycle progression was delayed at the S phase in nsp11-expressing cells compared to MARC-145 cells. A similar observation was recently made for coronavirus in cells expressing nsp15, which is a coronavirus homolog of PRRSV nsp11 [41]. In that study, SARS-CoV nsp15 was shown to downregulate the retinoblastoma (Rb) activity which is responsible for cell proliferation. As a consequence, a higher percentage of cells was accumulating at the S phase when expressing nsp15, supporting our observation of slower cell cycle progression in nsp11-expressing cells. The S phase tardiness may be associated with an altered DNA replication, since several MCM proteins, which are components of DNA helicase, were upregulated (Table 1). It is possible that the increase of helicase proteins might have caused a malfunction of the replication fork and thus the inhibition of DNA synthesis in MARC-nsp11 cells.

Virus-mediated cell cycle regulation is not uncommon and can be beneficial to viruses. In particular, it is true for DNA viruses replicating in the nucleus such as SV40, herpes simplex virus, and adeno-associated virus, in which by arresting the cell cycle of infected cells, the cellular DNA replicative machinery may be captured and utilized for viral DNA replication [42–44]. For RNA viruses, influenza virus replication has been shown to be regulated by helicase and the MCM complex consisting of MCM2-7 [45]. The interaction between the influenza virus PA polymerase and MCM complex increases the stability of RNA polymerase. In our study, MCM2, MCM4, and MCM5 were upregulated by nsp11. Even though the PRRSV replicates in the cytoplasm, the cell cycle regulation may be considered beneficial for the virus, since an available pool of cellular machineries can be maximally utilized towards the production of progeny at an early stage of infection. For PRRSV, nucleocapsid (N) protein has also been suggested to regulate the cell cycle progression by modulating the ribosomal RNA synthesis in the nucleolus [46]. Thus, it is possible that N and nsp11 may both regulate the cell cycle progression and facilitate virus production by targeting different cellular components modulating the host cell cycle. The N protein is an RNA-binding protein which contains the nuclear localization signal (NLS) and thus localizes in the nucleus and nucleolus. By yeast 2-hybrid screening, the inhibitor of MyoD family a (I-mfa) domain-containing protein was identified interacting with PRRSV N [47]. Since the I-mfa domain-containing protein interacts with cyclin T1 [48], which participates in the control of the cell cycle, this interaction suggests a regulatory role of N for the cell cycle [17]. Different from N, nsp11 resides in the cytoplasm and contains an endoribonuclease activity. Nsp11 may alter the function or expression of cytoplasmic cellular components such as mRNA modification, which may then result in the regulation of the cell cycle. Indeed, modification of cellular mRNA by nsp11 has been suggested previously [49]. In summary, our data show that the PRRSV nsp11 protein is responsible for the delay of the S phase and thus, together with N, may regulate the cell cycle progression. N functions in the nucleus and nsp11 functions in the cytoplasm.

In the current study, only a few immune-related cytokine genes were identified especially for IFN-related genes. This is probably due to the cell type and the treatments used in our study. MARC-145 cells are epithelial cells of the African green monkey kidney, and these cells are anticipated to produce only a minimal amount of cytokines, and their ability to produce IFN is limited unless they are stimulated. A study is in progress to compare the gene expression profiles in MARC-nsp11 cells before and after stimulation.

The microarray study allowed us to identify differential effects of the nsp11 protein on the cellular gene expression profiles. Studies are required to verify the significance of the differentially regulated gene expression. Analyses of relative protein modifications and activations especially for checkpoint proteins will help us understand the basis of the change of S phase caused by nsp11. Clearly, our data provide new insights into the understanding of cell-virus interactions and the pathogenic mechanisms of PRRSV and host responses to infection.

## Figures and Tables

**Table 1:** Five major cellular pathways regulated by PRRSV nsp11.

Pathway	Gene	Gene assignment	Transcript cluster ID	GenBank number	Fold change
Histone-related functions	HIST2H3D	Histone cluster 2, H3d	7919589	NM_001123375	2.48
	HIST1H2AI	Histone cluster 1, H2ai	8117580	NM_003509	2.69
	HIST1H2BH	Histone cluster 1, H2bh	8117426	NM_003524	2.31
	HIST1H2AK	Histone cluster 1, H2ak	8124524	NM_003510	3.66
	HIST1H2BK	Histone cluster 1, H2bk	8068898	NM_080593	1.98
	HIST1H2AI	Histone cluster 1, H2ai	8117583	NM_003509	2.31
	HIST1H4I	Histone cluster 1, H4i	8117537	NM_003495	1.84
	HIST1H2BM	Histone cluster 1, H2bm	8117594	NM_003521	2.13
	HIST2H2AA3	Histone cluster 2, H2aa3	7905079	NM_003516	1.94
	HIST2H3A	Histone cluster 2, H3a	7905085	NM_001005464	2.60
	HIST1H2AH	Histone cluster 1, H2ah	8117543	NM_080596	2.83
	HIST2H2BE	Histone cluster 2, H2be	7919637	NM_003528	2.06
	HIST1H3A	Histone cluster 1, H3a	8117330	NM_003529	2.00
	HIST1H3F	Histone cluster 1, H3f	8124437	NM_021018	3.81
	HIST1H2BB	Histone cluster 1, H2bb	8124394	NM_021062	2.14
	HIST1H2BI	Histone cluster 1, H2bi	8117429	NM_003525	1.95
	HIST1H3G	Histone cluster 1, H3g	8124440	NM_003534	2.95

Complement pathway	C1S	Complement component 1, s subcomponent	7953603	NM_201442	−2.93
	C1R	Complement component 1, r subcomponent	7960744	NM_001733	−1.93
	C3	Complement component 3	8033257	NM_000064	−2.19

MAPK signaling pathway	DUSP1	Dual specificity phosphatase 1	8115831	NM_004417	2.96
	FOS	FBJ murine osteosarcoma viral oncogene homolog	7975779	NM_005252	6.42
	MYC	V-myc myelocytomatosis viral oncogene homolog	8148317	NM_002467	1.96
	JUN	Jun protooncogene	7916609	NM_002228	2.02
	DUSP6	Dual specificity phosphatase 6	7965335	NM_001946	2.81
	SRF	Serum response factor (c-fos serum response element-binding transcription factor)	8119712	NM_003131	1.83

Proteasomal pathway	SUMO1	SMT3 suppressor of mif two 3 homolog 1	8058335	NM_003352	−1.62
	SNCA	Synuclein, alpha (non-A4 component of amyloid precursor)	8101762	NM_000345	−2.14
	PSMD3	Proteasome (prosome, macropain) 26S subunit, non-ATPase, 3	8006984	NM_002809	1.74
	PSMB10	Proteasome (prosome, macropain) subunit, beta type, 10	8002133	NM_002801	1.82
	PSMA7	Proteasome (prosome, macropain) subunit, alpha type, 7	8067382	NM_002792	1.81

DNA replication	MCM5	Minichromosome maintenance complex component 5	8072687	NM_006739	2.05
	MCM4	Minichromosome maintenance complex component 4	8146357	NM_005914	2.11
	MCM2	Minichromosome maintenance complex component 2	8082350	NM_004526	1.94

Cell cycle	CDC25A	Homo sapiens cell division cycle 25 homolog A	8086880	NM_001789	2.12
	CDC45	Cell division cycle 45 homolog	8071212	NM_001178010	2.00
	MYC	Homo sapiens v-myc myelocytomatosis viral oncogene homolog	8148317	NM_002467	1.96
	ORC1	Origin recognition complex, subunit 1	7916167	NM_004153	1.73

**Table 2:** Primer sequences for RT-qPCR for selected genes.

Primer	Primer sequence
TNFSF10-F	5′-AAGTGGCATTGCTTGTTTCT-3′
TNFSF10-R	5′-TTGATGATTCCCAGGAGTTTA-3′
DEPTOR-F	5′-TTTTGTGGTGCGAGGAAGTAAGC-3′
DEPTOR-R	5′-GCAGGACATTGAGCCCGTTG-3′
NOL6-F	5′-AACCGAGGACAGGAAAGGATTG-3′
NOL6-R	5′-TGTAGACCAGACTGAAAGGAGGC-3′
SH2-F	5′-TCTGTGAGTTTGAAGCCCTGAG-3′
SH2-R	5′-GCAATGTTTATCATCCCACCC-3′
EGR1-F	5′-AGCGATGAACGCAAGAGGCA-3′
EGR1-R	5′-GGATGGGTATGAGGTGGTGGC-3′
GAPDH-F	5′-CGGAGTCAACGGATTTGGTCGTA-3′
GAPDH-R	5′-AGCCTTCTCCATGGTGGTGAAGAC-3′
